# Use, Perceptions and Attitudes of Cognitive and Sports Performance Enhancing Substances Among University Students

**DOI:** 10.3389/fspor.2022.744650

**Published:** 2022-04-12

**Authors:** Demitri Constantinou, Ivan Aguiyi

**Affiliations:** ^1^Centre for Exercise Science and Sports Medicine, Faculty of Health Sciences, University of the Witwatersrand, Johannesburg, South Africa; ^2^International Federation of Sports Medicine (FIMS) Collaborating Centre of Sports Medicine, Johannesburg, South Africa

**Keywords:** doping, stimulants, methylphenidate, anti-doping, university students, neuroactive agents

## Abstract

The use of prohibited performance enhancing substances (PESs) is well-documented among athletes, and includes student athletes in institutions of higher learning. In addition to using PESs to enhance sporting performance, they may be used for cognitive and academic achievement, specifically through the use of neuroactive substances. The latter in particular is under-researched and poses public health risks. Understanding the extent and reasons for using or not using PESs by university students will assist in addressing further research, and means to deter their use. This study aimed to explore the prevalence, attitude, and perceptions of the use of both sport and academic performance-enhancing substances by students at two universities in Johannesburg, South Africa. The study utilized a cross-sectional, descriptive survey design using a self-administered online questionnaire. All registered students were invited to participate via email using the university communications modes, with a link to a Google Forms questionnaire. Ethical approval was granted for the study and data were collected anonymously. The sample size of responses with completed data was 548, comprising predominantly female and undergraduate students, with 32 (6%) indicating current or past use of PESs. Among the prohibited substances used, neuroactive drugs were mostly used, with cannabis the highest followed by stimulants. The most popular reasons reported were for academic and cognitive performance. Most responded with what would be deemed ethically appropriate answers on the perception of PES use and 72% of the participants believe that a drug-testing program will prevent their use in the university, and further, that educational programs will help improve students' knowledge of PES. Although relatively low usage, university students use performance enhancing substances, mostly for cognitive enhancement using neuroactive stimulants. Most believe that the use of all forms of PESs is high among their peers; and favor education and testing programs, suggesting that intervention programs may be effective. Better defined research should be conducted to unpack the broad findings in this study.

## Introduction

Doping has been defined as the use of prohibited drugs, methods, or other substances for performance enhancement. It occurs in all age groups, genders and at all levels of sport competition (Fernandez and Hosey, 2009). The use of PESs among athlete populations is to gain a competitive advantage. The World Anti-Doping Agency (WADA) definition of doping applies to competitive athletes and is defined as the occurrence of one or more of the 13 anti-doping rule violations (ADRV) of the World Anti-Doping Code (WADC). These ADRV's includes the “Use or Attempted Use by an Athlete of a Prohibited Substance or a Prohibited Method” (World Anti-Doping Agency, 2021).

The WADA publishes an annual list of prohibited substances and methods (World Anti-Doping Agency, 2021), recognized by all WADC signatories. The prohibited substances, and rules, that elite or competitive athletes are subjected to with respect to anti-doping rule violations and sanctions are also governed by WADA. However, doping in sports or the use of PESs is not only observed in professional athletes (Striegel et al., 2010), but also in recreational athletes (Striegel et al., 2006) who may have different motives for using PESs compared to elite athletes (Barkoukis et al., 2013; Chirico et al., 2021). Although the Anti-Doping Rules may apply to recreational level athletes (South African Institute for Drug-Free Sport, 2021), they may face similar or less severe sanctions, if at all, for using PESs (South African Institute for Drug-Free Sport, 2021).

In addition of the use to enhance sports performance, there is a growing trend to use substances that enhance cognitive function for academic performance, attention, memory, or mood. Students use these with the intention to optimize study duration, concentration, improve attention span, and also decrease anxiety (De Santis et al., 2008; Rabiner et al., 2009). Cognitive doping or “neuro-enhancement” (the use of drugs to improve cognitive performance) in the absence of any medical need is gaining attention (Franke et al., 2013), and is of concern (Maher, 2008; Striegel et al., 2010; Dietz et al., 2013). Cognitive doping may include using illicit substances (e.g. cocaine) and prescription drugs such as stimulants (e.g. amphetamines and methylphenidate) (Franke et al., 2011b), which are commonly used for conditions such as Attention Deficit Hyperactivity Disorder/Attention Deficit Disorder (ADHD/ADD) and narcolepsy. Beyond the cognitive doping effects, they have the potential for physical performance enhancement and in most countries are available to patients only with a physician's prescription. University students diagnosed with ADHD/ADD may legitimately use such stimulants with the therapeutic intention of normalizing a diagnosed medical condition. However, using such stimulants for non-medical purposes poses significant public health risks and it has been recommended that tertiary institutions include non-medical use of stimulants in their policies of academic integrity (Faraone et al., 2020). There may be those that abuse these substances to promote academic advantage (Cakic, 2009), such as improved memory and less sleep with the intention of increasing study time (Teter et al., 2006; Cakic, 2009). Studies of amphetamines use in high school and/or university students report that prescription drug use for cognitive enhancement was common among American and German students (Teter et al., 2006; Wilens et al., 2008; Franke et al., 2011a). A Swiss-based study showed a lifetime prevalence rate of 6.2% by students for non-medical use of prescription stimulants (methylphenidate, amphetamine, and/or modafinil) (Ott and Biller-Andorno, 2014).

University students all have the common objective of academic success, and some may also be student athletes where peak sporting performance is also a goal. This may place them at increased risk for substance abuse (Wechsler et al., 1997). This poses a number of scenarios: (a) student athletes that use PESs for sporting performance; (b) non-athlete students that use neuroactive drugs (stimulants and/or cannabinoids) for cognitive performance; (c) student athletes that use PESs, including neuroactive drugs for academic benefit; and (d) student athletes that use neuroactive drugs for cognitive and academic benefit (and not intentionally for sporting performance). The latter, if are also elite athletes, and fall within a WADA or national anti-doping agency registered testing pool (South African Institute for Drug-Free Sport, 2021), have the potential of unintentionally falling foul of anti-doping regulations in sport. Students that are neither elite or recreational athletes but use PESs for cognitive enhancement would not face anti-doping rule violation sanctions, however, depending on the substances used, may be subjected to institutional disciplinary or even criminal charges.

Few studies on PESs use have been conducted in young athletes at school level and even fewer in university students. A Swedish study on school students reported that a single trial of performance-enhancing substance use occasionally reached 15% (Kindlundh et al., 1998), and high school boys in Johannesburg, South Africa reached as much as 30% (Gradidge et al., 2011). Motives for the use include pressure from peers, teammates, coaches, and sometimes families; and a desire to win at all costs (Gradidge et al., 2011; Pappa and Kennedy, 2013; Yager and O'Dea, 2014).

Prevalence data in United States of America university students of cognitive enhancing substance use estimates stimulant use between 5 and 35% (Smith and Farah, 2011), and among United Kingdom students, <10% lifetime prevalence use has been reported (Singh et al., 2014). Among Swiss university students 4% reported using methylphenidate, and entertained the idea that cognitive performance enhancement was acceptable (Maier et al., 2013). Although a low prevalence rate of use was observed among German students, 80% of the study participants stated that they would consider using stimulants (Franke et al., 2011a). In an Australian survey, 2.4% of university students reported using academic enhancing substances despite most expressing concern about their efficacy and potential adverse effects (Mazanov et al., 2013).

Avois et al. as far back as 2006 reported that doping with central nervous system stimulants poses significant public health risks and preventive measures should be proactively pursued (Avois et al., 2006). They reported that since 1988 International Olympic Committee accredited laboratories have reported on positive tests for stimulants which is now the second most commonly used PESs after anabolic androgenic steroids (World Anti-Doping Agency, 2020). Of all stimulants, methylphenidate is the most commonly used (World Anti-Doping Agency, 2020). To date the use of PESs and CNS stimulants by university students in South Africa is yet to be documented, which together with student attitudes and perceptions, is salient to address.

The knowledge and research gap was identified as being the use of PESs by university students, either for sport or cognitive doping. The use of substances prohibited in sport according to the WADA Prohibited List was the ADRV point of reference explored in this study.

This is the first such study that we are aware of conducted in university students in South Africa, and aimed to explore the use, behavioral attitude and perceptions of the use of sport and neuroactive performance enhancing substances.

## Materials and Methods

### Study Design and Site

This study utilized a cross-sectional, descriptive survey design and was carried out in Johannesburg, South Africa at two universities; both which were part of the same anti-doping program endorsed by the South African Institute for Drug Free Sport. Registered students over the age of 18 years were included, with no exclusion criteria. Due to the nature of research protocol at the universities, invitations to participate were sent to the students by the university registrars' offices using their institutional databases and email communication mechanisms.

### Measurement Tool

Students over the age of 18 years were invited to participate and those who agreed completed a previously used and validated self-administered online questionnaire (Gradidge et al., 2011) using Google forms (Google Surveys). The questionnaire was divided into five sections with background information and demographics, general perceptions of substance use, general knowledge of PESs, use of and reasons for use of PESs, and attitudes of use. A pilot study with 12 students was done to test the questionnaire reliability using Cronbach's alpha statistical method to ascertain the internal consistency of the research tool.

The email invitations had a link to the questionnaire which the students could access on agreeing to participate, and the data were summated as a function of the forms and accessed by the researchers.

### Ethical Consideration

Data were collected anonymously. Ethical clearance was applied for and granted by the first university (clearance number M1911176) and based on that, the second university issued a clearance letter for the study. The necessary university permissions to target students were sought and granted by the relevant university authorities.

## Results

Descriptive data were group-analyzed using Stata 14.2 statistical software package (StataCorp. 2015. *Stata Statistical Software: Release 14*. College Station, TX: StataCorp LP). Open-ended questions were clustered into themes of response.

### Study Population and Demographics

The total population was all students who were registered and numbered over 50,000. It is assumed all would have received the communication from the universities, and had access to the questionnaire. A total of 644 responses were received of which 96 were excluded due to incomplete or improper responses (responses total *n* = 548).

The age range was 18 to 40 years, with most (70%) between the ages of 18 to 24 years. Of the 548 participants, 58% were female and 40% were male and 2% preferred not to disclose their gender. Fifty-seven percent of the participants were active in sports with the other 43% not indicating any sports participation. Of those that participated in sport, the most popular sport was soccer (8%), followed by athletics (running) (5%), swimming (2%), netball (2%), rugby (1%), basketball (1%), hockey (1%), boxing (1%), tennis (1%), and all others sports (16%). About 20% engaged in more than one type of sport.

### Sources of Information on Performance-Enhancing Substance (PES) Use

Most participants reported not ever accessing PESs information (47%) and about a third (30%) sourced information from multiple sources. The most common single source was the Internet (9.3%).

### Patterns of Performance-Enhancing Substance (PES) Use

**Figure 1:**
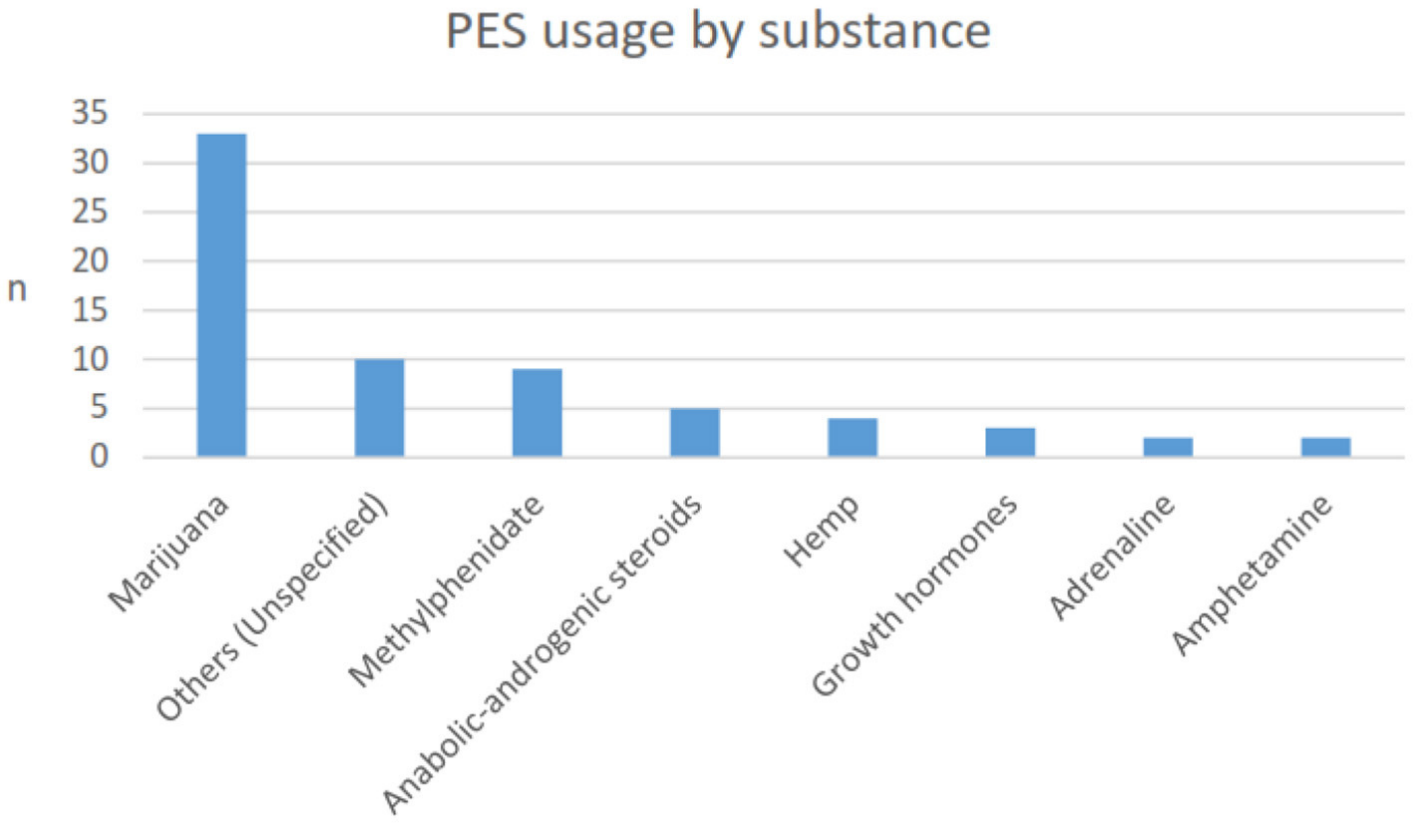
Types of PES substances used. *N* = 68, as the 32 respondents may have used more than one substance.

**Figure 2:**
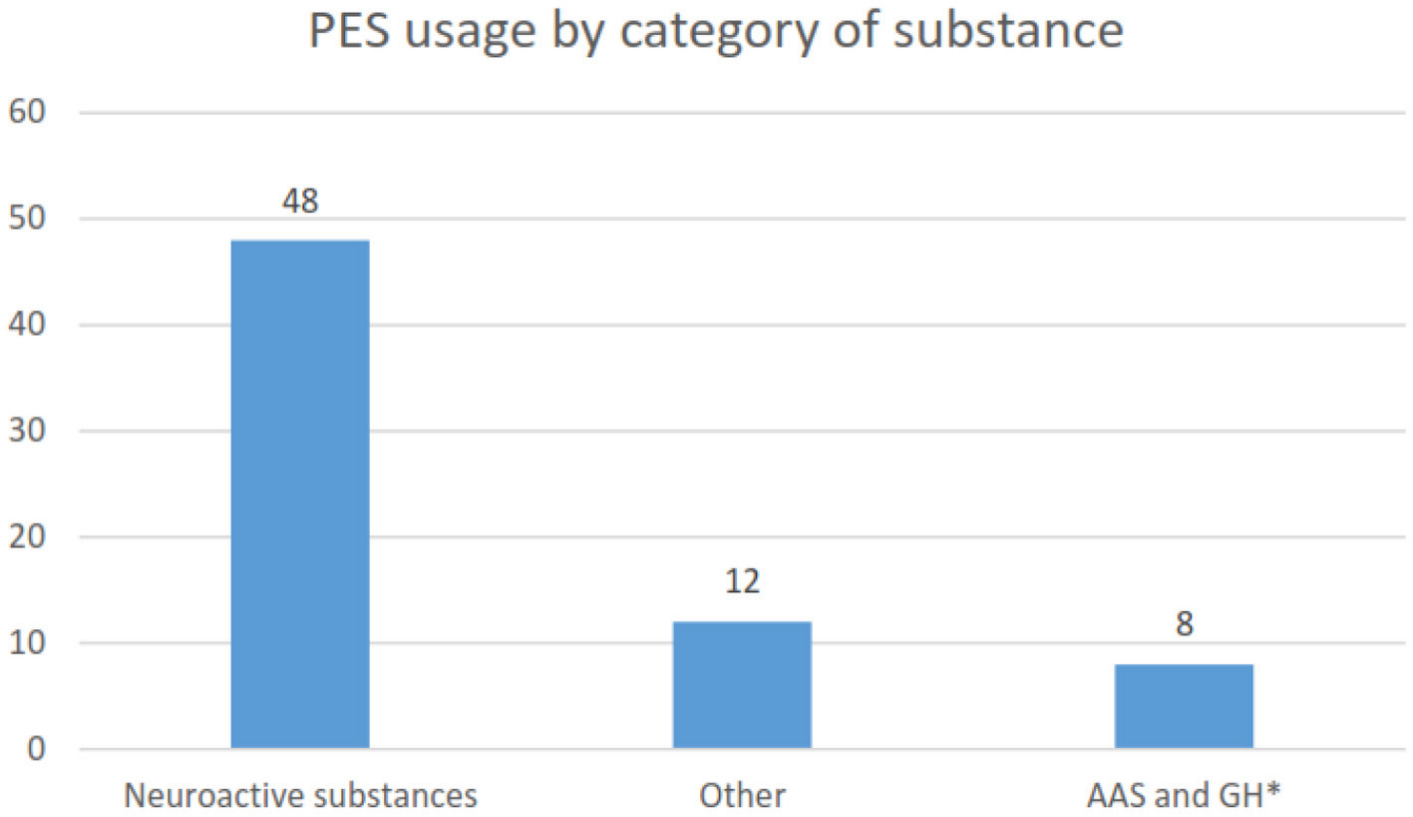
Grouped results of types of substances used. AAS, anabolic androgenic steroids; GH, growth hormone.

The majority of participants had reported never having used PESs (94%). Of the 32 (6%) that reported current or past use, nine (2%) reported current use, and 23 (4%) indicated previous use. Approximately 4% reported using PES for academic (cognitive) purposes, 2% for sport, and 1% for both sport and cognition. Two-thirds (66.7%) of those that had previously used, or were currently using PES, stated they had started this practice whilst at university. Cannabis was the most commonly used neuroactive substance (56%), and methylphenidate use comprised 1.3% (Figure 1), which could have been be used for the medical conditions of ADHD/ADD and/or cognitive performance. Use of stimulants was not segregated between use for a medical condition or not. Figure 2 illustrates the use of neuroactive PES being the highest as compared to the other substance categories.

### Reasons for Using Performance Enhancing Substances (PES)

**Figure 3:**
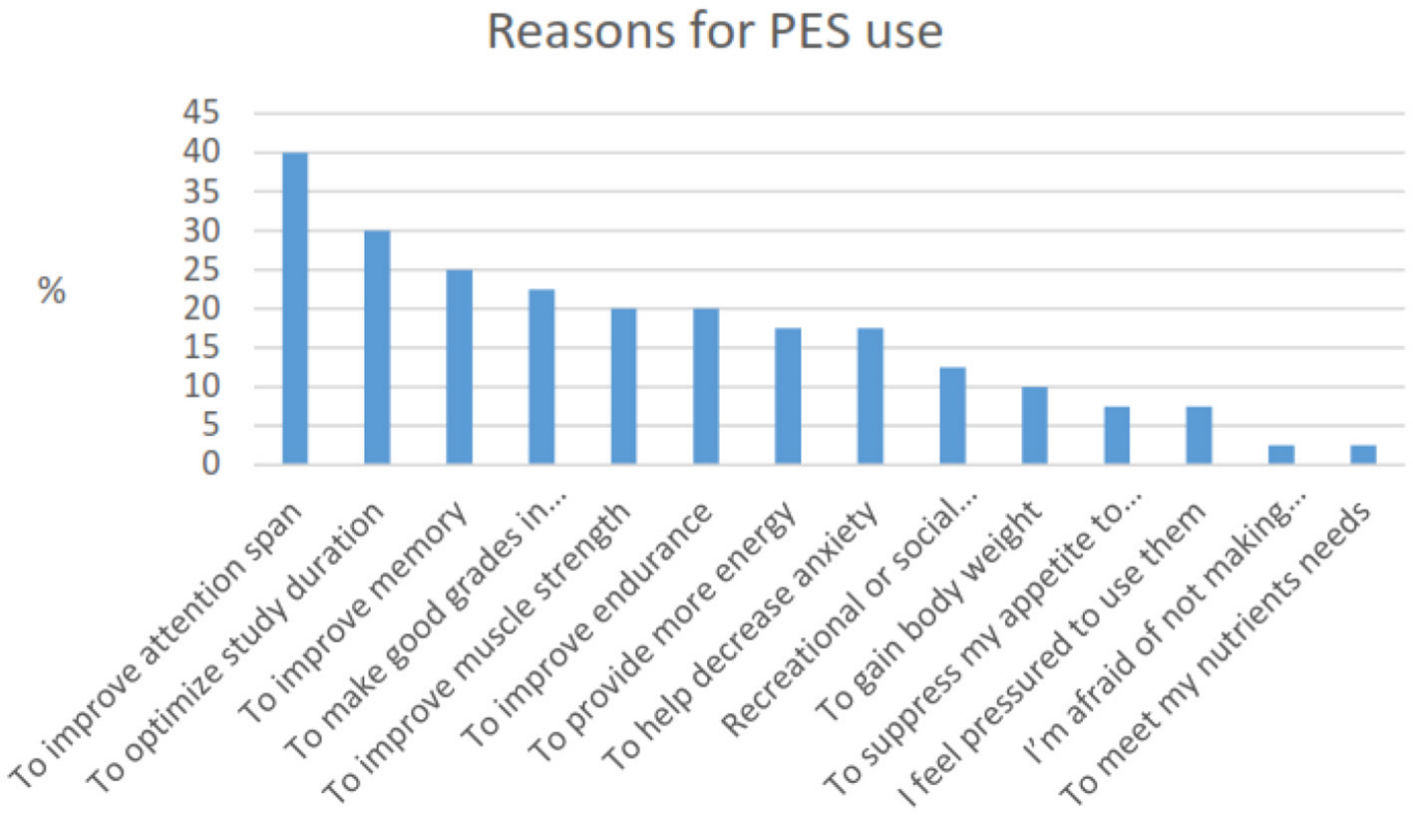
Reasons for PES substance use.

**Figure 4:**
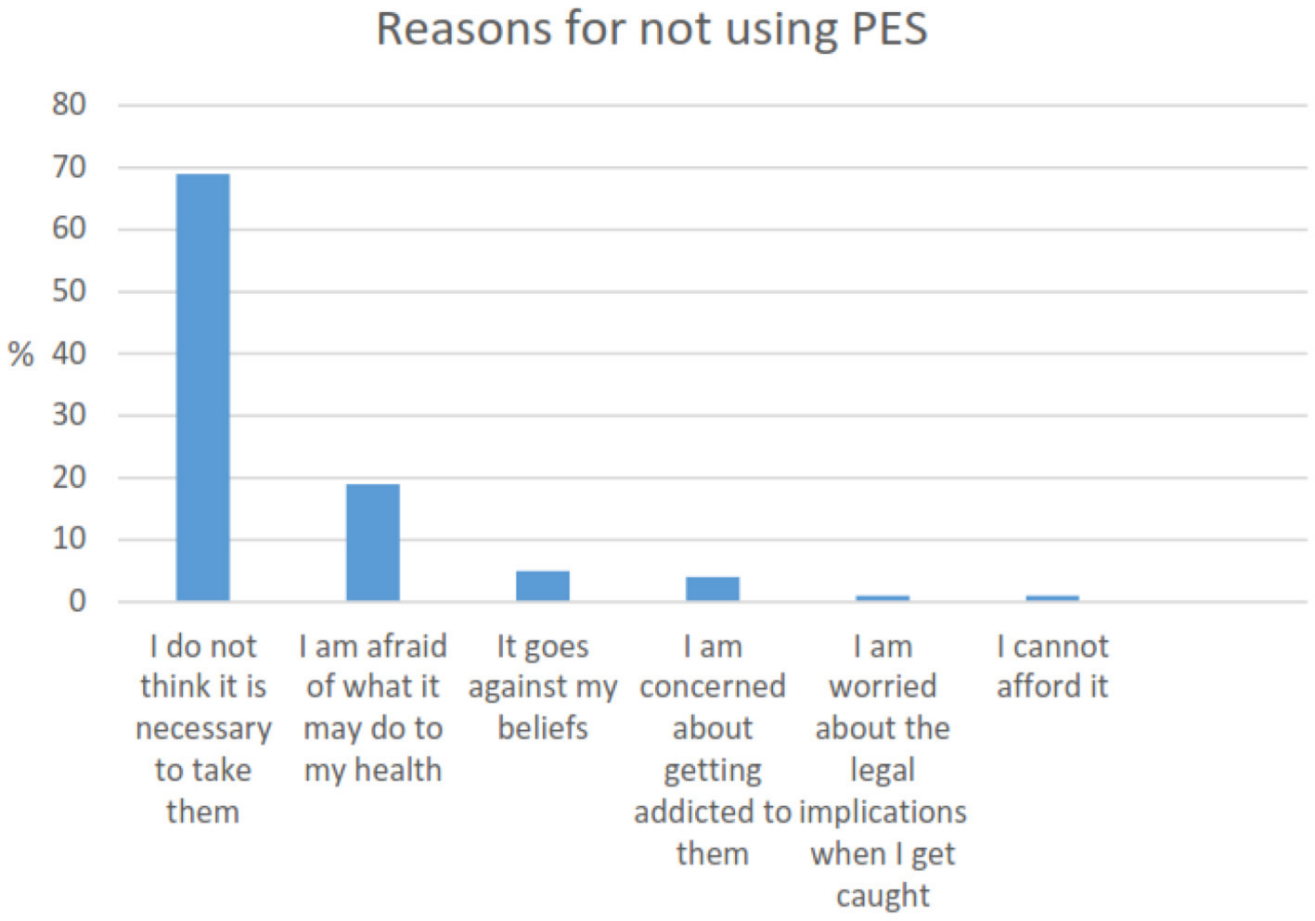
Reasons for not using PES substances.

The survey cast a wide net that included questions on the reasons for using PES that encompassed physical and cognitive performance as these were unknown. The most prevalent reason for using PES among the university students was to improve attention span (40%). Other reasons (Figure 3) included to optimize study duration (30%), improve memory (25%), make good grades (22.5%), and improve strength and endurance (20%). We further inquired of those that did not use PES their reasons for not using (Figure 4). The most popular reason cited for not using PES was “I do not think it is necessary to take them.”

### Attitudes and Perceptions of University Students Toward Performance Enhancing Substances (PES) Use

**Figure 5:**
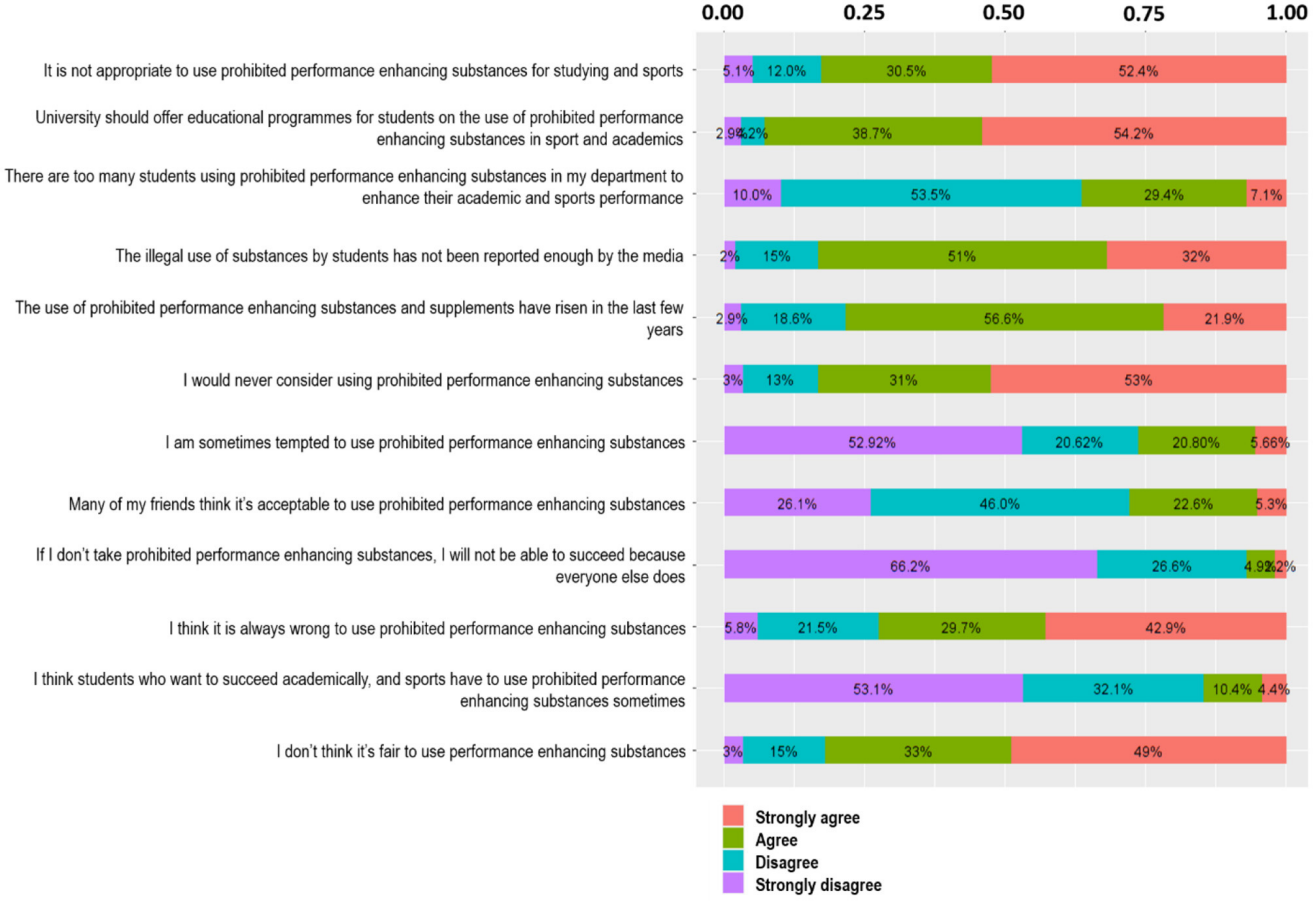
Details of attitudes and perceptions of university students toward performance enhancing substances (PES) use.

The general attitudes and perceptions of the participants toward PESs use are summarized in Table 1, with Figure 5 providing more detail on individual responses. The majority of participants (73%) believe that prohibited PESs were being used by fellow students and that this is increasing; but that a drug-testing program will prevent the use of PESs (72%). Seventy five percent indicated that they thought it unethical for students to use prohibited PESs for either sport or academic purposes. More than half (57%) felt students were being pressured to use PESs.

**Table 1:** Attitude and perceptions toward PES use (*n* = 548).

**Questions**	**No n (%)**	**Yes n (%)**
Do you think the use of prohibited performance-enhancing substances in sport is increasing?	133 (24)	415 (76)
Do you think that the use of prohibited performance-enhancing substances in sports or for studying is unethical?	138 (25)	410 (75)
Do you think prohibited performance-enhancing substances are used by students?	148 (27)	400 (73)
Do you think that a drug-testing program will prevent the use of prohibited performance-enhancing substances in the university?	152 (28)	396 (72)
Do you think students are being pressured to use prohibited performance-enhancing substances? (By coaches, friends, their parents, etc.)	233 (43)	315 (57)

Results indicated that the prevalence of PESs and neuroactive substance use among the participants was 6%, with 4% using for cognitive performance enhancement and 1% for both cognitive and sport enhancement. The most used substance was cannabis, and methylphenidate the most used stimulant. Over two-thirds believe that a drug-testing program will prevent the use of PESs in the university and educational programs will help improve the knowledge of students.

## Discussion

A broad exploratory survey was undertaken to assess the as-yet unknown behaviors and attitudes of physical and cognitive enhancing substance use among South African university students, and to define future research.

### Demographics

Six hundred and forty-four students from Johannesburg-based universities volunteered to participate in the online survey, of which 548 had complete data. Most were undergraduate students (63%) aged between 18 and 24 years, and female (58%). This is in keeping with the demographic distribution in the universities (University of Johannesburg, 2020; University of the Witwatersrand, 2021).

### Prevalence of Common Cognitive and Sports Performance Enhancing Substance (PES) Used by Participants

The use of PES for sport, cognitive and recreational purposes is widespread and of concern (Singh et al., 2014). It has been reported that in competitive sports, prevalence rates can substantially differ (Gleaves et al., 2020). Our cohort were not competitive athletes and although 6% reported using PES either currently or previously for a variety of reasons, the majority indicated that they had started using at university (within the preceding 1–3 years).

Motivation for using PES differs amongst different groups, with competitive athletes seeking enhanced physical and sporting performance. In cohorts similar to our study, there may be use of substances for both performance and recreational purposes (Madu and Matla, 2003; Rottcher, 2006; Kohler et al., 2010). Our study showed the most used substance was cannabis [> 0.3% of tetrahydrocannabinol (THC)], probably for recreational purposes and not performance enhancing, although it has been used with the belief that it enhances cognitive function for academic purposes. A study conducted among high schools in a rural area in South Africa showed that 17% of male adolescent learners used cannabis (Taylor et al., 2003) and another study reported as many as 39% of young people use cannabis (Rottcher, 2006). Hemp use was much less and are plants defined as having ≤0.3% THC, with very little psychoactive effect (Ferguson, 2020). A study conducted among French adolescents found cannabis use was quite widespread, with some of the respondents admitting its use for cognitive enhancement (Lorente et al., 2005).

Another French study reported that more than 50% of first-year medical students used cannabis during preparation for exams (Laure, 2000) and yet university sports science students used cannabis to enhance both non-sports (36%) and sports (12.5%) performance. As such the use of cannabinoids in our study is reflective of local community trends, and also worldwide use by university students.

Of concern is that young people who show higher tendencies for hazardous social behavior such as the use of illegal drugs for recreational purposes may be more susceptible to using prohibited PESs both for sport and cognitive performance, particularly at tertiary institutions (Laure et al., 2004). Interestingly about two-thirds in our study reported having started using at university level. This emphasizes the need to deeper address the “why” and “how” to develop effective preventive programs and strategies.

### Attitude and Perceptions Toward Performance Enhancing Substance (PES) Use

Our findings included that over two-thirds of the participants suspect other students commonly use these substances yet believed it is unethical for university students to use for either academic or sport enhancement. Further, most felt it an unfair practice. Reasons for using PESs included the perception that if they did not use these substances, they would not be able to perform adequately, and also because it is a common practice. Direct pressure was cited; and most felt that students were being pressured by coaches, friends, and even family members to use these substances. Consideration must be given to address not just students/athletes in anti-doping programs for both sport and cognitive doping, but also beyond, directed at those sources of pressure. Self-image is a factor at university level too, with social status and acceptance among youths and university students deemed important. This may influence the use of PESs, spanning from physical image through to indulging in risky behaviors.

Knowledge regarding PESs varied, and half of the participants felt media information is insufficient, and as many felt the university should offer educational programs to students on the use of prohibited PES for both sports and academia. Most also felt that dope testing in university students would be a deterrent, in keeping with a study from the United Kingdom (Whitaker et al., 2014).

A unique approach we took was to ask questions on why the participants did not use PESs, providing another angle of insight. Overall the majority of answers in all attitude questions returned responses that would be deemed ethical and in support of anti-doping. This is promising and together with the suggested interventions of education and testing, informs an approach for anti-doping programs aimed at university level students.

### Study Limitations

Despite being the first study of its kind including the use of PESs for cognitive and sports purposes at university level, there are limitations. As with all self-reported surveys, participants may have not always been truthful in their response and may have expressed certain biases. The questionnaire itself in order and questions was based on a previously used survey for a similar but different population. Despite a sizeable sample of 548, the response rate is relatively low if one consider there were likely over 50,000 registered students at the time of the study; and in turn the participants that used PESs was relatively low. Incidence and prevalence were not differentiated. Use of stimulants was not segregated between use for a medical condition or not. Analysis grouped all genders, is descriptive and has not included correlation or regression analyses. These limitations will be considered in future studies.

## Conclusions

Our study confirmed that of the relatively low numbers of students that use PESs at university, neuroactive substance use was the highest with the major reason being for cognitive enhancement. Cannabis was the most frequently used neuroactive substance, followed by methylphenidate. Students believe use is higher than actual use among their peers, and that there is pressure from external sources for this. The attitudes reflect positive ethical beliefs and together with education and testing programs, can inform interventions to address the use of PESs in university students.

### Recommendations

The study provided valuable information, and based on the findings, we recommend:

(a) Based on the current findings, focused future research should be conducted. Similar surveys should use an updated and validated questionnaire, which uses logical flow programming to better differentiate domains.
(b) Education programmes by experts from education and social units within the universities and input from the national Anti-Doping organizations and other relevant stakeholders to enhance knowledge—starting at school level with adolescents, and intensified at university level.
(c) Education extended to coaches, family and friends, who possibly exert pressure on students,
(c) Dope-testing programs aimed at university student athletes.

## Data Availability Statement

The raw data supporting the conclusions of this article will be made available by the authors, without undue reservation.

## Ethics Statement

The studies involving human participants were reviewed and approved by Human Research Ethics Committee (Medical) of the University of the Witwatersrand. The patients/participants provided their written informed consent to participate in this study.

## Author Contributions

DC conceptualized the design of the study and finalized the manuscript. IA organized the database and performed the statistical analysis. IA and DC wrote the first draft of the manuscript and sections. Both authors contributed to manuscript revision, read, and approved the submitted version.

## Conflict of Interest

The authors declare that the research was conducted in the absence of any commercial or financial relationships that could be construed as a potential conflict of interest.

## Publisher's Note

All claims expressed in this article are solely those of the authors and do not necessarily represent those of their affiliated organizations, or those of the publisher, the editors and the reviewers. Any product that may be evaluated in this article, or claim that may be made by its manufacturer, is not guaranteed or endorsed by the publisher.
